# Review of recent taxonomic changes to the emerald moths (Lepidoptera: Geometridae: Geometrinae)

**DOI:** 10.3897/BDJ.8.e52190

**Published:** 2020-04-30

**Authors:** David Plotkin, Akito Y. Kawahara

**Affiliations:** 1 Department of Entomology and Nematology, University of Florida, Gainesville, United States of America Department of Entomology and Nematology, University of Florida Gainesville United States of America; 2 Florida Museum of Natural History, Gainesville, United States of America Florida Museum of Natural History Gainesville United States of America

**Keywords:** Classification, Geometridae, Geometrinae

## Abstract

**Background:**

The subfamily Geometrinae (Lepidoptera: Geometridae), commonly known as emerald moths, is an ecologically diverse group of moths with over 2,500 described species. Many taxonomic and phylogenetic studies of Geometrinae have been undertaken in the past decade, resulting in hundreds of new taxonomic changes since online publication of the most recent checklist in December 2007.

**New information:**

This review synthesises the last 12 years of alpha-taxonomic research in Geometrinae. A comprehensive list of Geometrinae genus- and species-group descriptions, synonymies, combinations and other taxonomic changes, made since 2007, is provided. Since 2007, the known species richness of Geometrinae has increased from 2,529 to 2,642 species; an updated list of all these species is presented in a supplementary spreadsheet.

## Introduction

The family Geometridae is an incredibly diverse lineage of moths that contains over 23,000 described species, making it the second-most speciose family in all of Lepidoptera (Scoble and Hausmann 2007, van Nieukerken et al. 2011). Although the subfamily Geometrinae only comprises roughly one-tenth of this species richness, it is one of the more recognisable geometrid subfamilies because of the green colouration found in most adults and some larvae. Geometrinae are consequently known as ‘emerald moths’ and have been the subjects of studies on phenotypic plasticity and polyphenism (Greene 1989, Canfield et al. 2008). Many authoritative taxonomic works on Geometrinae have been published over the years, but with many new species being described annually, it does not take long for an update to become necessary.

Parsons et al. (1999) published a two-volume catalogue of the geometrid moths of the world, which is currently the most recently printed work that contains a comprehensive checklist of the global emerald moth fauna. However, between 1999 and 2007, one of this checklist’s co-authors, Malcolm Scoble, worked with Axel Hausmann to update the checklist; these revisions are hosted on the Lepidoptera Barcode of Life website. At the time of the most recent update (December 2007), the online checklist contained 269 genera and 2,529 species of Geometrinae. Since then, hundreds of taxonomic changes have been made in this subfamily, including over 100 new species. In this review, we catalogue the last 12 years of emerald moth taxonomy and update the list of geometrines provided by Scoble and Hausmann (2007) to include all newly-described emerald moth species.

## Materials and methods

Updates and changes to the online checklist of Geometrinae are presented in alphabetical order by genus, following the format used by Scoble and Hausmann (2007). Since their checklist was last updated in December 2007, the taxonomic literature published between January 2008 and December 2019, inclusive, was consulted. It was also found that some taxonomic works (e.g. Beljaev 2007) were published prior to December 2007, but were not incorporated into the update; these are consequently included in this review.

Within each genus section, valid species names are listed in alphabetical order, with junior synonyms placed on an indented line following the corresponding senior synonym. Only genus- and species-group taxonomic changes in Geometrinae are discussed here; a review of recent family-group taxonomic changes can be found in Ban et al. (2018) and Murillo-Ramos et al. (2019), both of whom have also proposed new taxonomic changes to the geometrine tribes and subtribes, based on molecular phylogenetic data. Scoble and Hausmann (2007) did not include subspecies in their checklist, but post-2007 taxonomic changes to subspecies are discussed in this review.

The type of taxonomic change is indicated in parentheses. In this catalogue, the word 'new' and the abbreviation 'nov.' (novus, -a, -um) both denote that a taxonomic change was recent enough to not appear in the checklist of Scoble and Hausmann (2007). In this review, no taxonomic changes are proposed for the first time; this is further emphasised by the use of quotation marks surrounding each record of taxonomic change in the Results. Citations for recent taxonomic changes are provided in corresponding Remarks sections for each genus. The vast majority of taxonomic changes discussed here were proposed based solely on morphological evidence, such as variation in colour patterns, wing venation and genitalic characters of the adults. If molecular evidence were used to justify a taxonomic change, this is noted in the corresponding Remarks section.

If the status of a subspecies has been changed or a new synonymy has been proposed, the name of the associated valid species name is provided for context. Otherwise, species that have not undergone any taxonomic changes since the publication of Scoble and Hausmann (2007) are not included in the main text; a full list of all current Geometrinae species names is provided in the supplementary material. Similarly, synonyms that are not directly associated with a recent taxonomic change are excluded from the text.

Multiple Latin abbreviations for standard taxonomic terms are used throughout the text. Since the abbreviations themselves are not standardised across all taxonomic literature and do not appear at all in Scoble and Hausmann (2007), the notation used by Kitching et al. (2018) for a recent checklist of bombycoid moths was applied here in both the main text and supplementary material. These abbreviations and their definitions, are as follows:

“comb. nov.” – new combination

“comb. rev.” – revived combination

“gen. nov.” – new genus

“nom. nov.” – new replacement name

“nom. nud.” – nomen nudum (without description, thus unavailable)

“sp. nov.” – new species

“ssp. nov.” – new subspecies

“stat. nov.” – new status

“stat. rev.” – revived status

“syn. nov.” – new synonym

“syn. rev.” – revived synonym

## Data resources

The list of taxonomic changes made in Geometrinae since publication of Scoble and Hausmann (2007) and the updated list of emerald moth species of the world are provided as tables (Excel format) in Suppl. material 1.

## Checklists

### List of Geometrinae genera and species associated with recent taxonomic changes

#### Acidaliastis

Hampson, 1896

299EF5A0-BD51-546F-99E0-802C91EA1ADC

Acidaliastis porphyretica Prout, 1925
Acidaliastis subbrunnescens Prout, 1916

##### Notes

The AfroMoths database (De Prins and De Prins 2019) states, without a citation, that *Acidaliastis porphyretica* was transferred to the genus *Acidromodes* Hausmann, 1996 and that *Acidaliastis subbrunnescens* was transferred to *Hemidromodes* Prout, 1916. After searching the literature, these names were found on other online species lists, but there did not appear to be any formal publications that proposed these new combinations. Thus, *Acidaliastis porphyretica* and *Acidaliastis subbrunnescens* are currently considered the valid names for these species.

#### Agathia

Guenée, [1858]

77C41F2B-6DDF-5CB0-98B4-E155DB33EA4B

Agathia microlaetata Goyal, Kirti & Saxena, 2018 ("sp. nov.")

##### Notes

The name and locality of *Agathia microlaetata* appeared in Kirti et al. (2012), but this new species was not formally described until it appeared in Goyal et al. (2018).

#### Albinospila

Holloway, 1996

B10F57EE-5614-51E3-916B-0750ACF3A7C5

Albinospila juancarlosi Tautel & Barrion-Dupo, 2017 (“sp. nov.”)
Albinospila variifrons (Prout, 1917) (“comb. nov.”)

##### Notes

One new species was described (Tautel and Barrion-Dupo 2017). *Albinospila variifrons* was transferred from *Comostola* Meyrick, 1888 by Tautel and Barrion-Dupo (2017).

#### Aoshakuna

Matsumura, 1925 (“stat. rev.”)

3830F8B2-D432-5631-B602-75E0E57D3A42

Nipponogelasma Inoue, 1946 (“syn. nov.”)
Aoshakuna lucia (Thierry-Mieg, 1916) (“comb. nov.”)
Aoshakuna sachalinensis Matsumura, 1925 (“syn. nov.”)
Aoshakuna lucia ussurica Beljaev, 2007 (“ssp. nov.”)

##### Notes

One new subspecies was described (Beljaev 2007).

*Aoshakuna* was previously a junior synonym of *Chlorissa* Stephens, but was re-instated by Beljaev (2007). In the same revision, Beljaev (2007) subsequently designated *Nipponogelasma* a junior synonym of *Aoshakuna.* As a result of this synonymy, *Nipponogelasma lucia* was transferred to *Aoshakuna*, creating the new combination *A. lucia*. Beljaev (2007) then synonymised this species with *A. sachalinensis*, the type species of *Aoshakuna*.

#### Assachlora

Viidalepp & Lindt, 2012 (“gen. nov.”)

C54EDD5E-CB5A-5F51-9575-D5E3B0DD1C8C

Assachlora assa (Druce, 1892) (“comb. nov.”)
Assachlora julietae Viidalepp & Lindt, 2012 (“sp. nov.”)
Assachlora mitigata (Prout, 1912) (“comb. nov.”)

##### Notes

One new species was described in this new genus (Viidalepp and Lindt 2012). *Assachlora assa* and *A. mitigata* were transferred from *Phrudocentra* Warren, 1895 by Viidalepp and Lindt (2012). *Assachlora* currently contains three species, with *A. assa* designated as the type species.

#### Bathycolpodes

Prout, 1912

F9BDDDDF-7B4F-5050-9DF7-DE51779BE45B

Bathycolpodes parexplanata Karisch & Hoppe, 2010 (“sp. nov.”)
Bathycolpodes roehrichti Karisch, 2010 (“sp. nov.”)
Bathycolpodes scheeli Karisch & Hoppe, 2010 (“sp. nov.”)
Bathycolpodes subferrata Prout, 1930 (“stat. nov.”)
Bathycolpodes subfuscata (Warren, 1902)

##### Notes

Three new species were described (Karisch 2010). Although Henri Hoppe is credited with co-authorship of the new species *Bathycolpodes parexplanata* and *B. scheeli* in Karisch (2010), he is not credited as an author of the publication.

The subspecies *Bathycolpodes subfuscata subferrata* was elevated to the species *B. subferrata* by Karisch (2010).

#### Bustilloxia

Expósito, 1979

893DA29D-F9BF-541D-9A6C-DDC7BE3BDE2C

Bustilloxia saturata (Bang-Haas, 1996)
Bustilloxia saturata iberica Hausmann, 1995 (“syn. nov.”, followed by “stat. rev.”)

##### Notes

Leraut (2009) changed the status of *Bustilloxia saturata iberica* from a subspecies to a junior synonym of *B. saturata*. Müller et al. (2019) later revived *B. s. iberica* as a valid subspecies.

#### Chlorissa

Stephens, 1831

9CF0591E-8492-50C5-91C5-D863DBBD43D9

Chlorissa archipelago Tautel, 2016 (“sp. nov.”)
Chlorissa obliterata (Walker, 1863) (“syn. nov.”, followed by “stat. rev.”)
Chlorissa viridata (Linnaeaus, 1758)

##### Notes

One new species was described (Tautel 2016). Leraut (2009) synonymised *Chlorissa obliterata* with *C. viridata* and Müller et al. (2019) subsequently revived its status as a valid species.

#### Chloristola

Holloway, 1996

31A7DDCD-6C4E-5772-A26B-8CA41D66DAAF

Chloristola murzini Tautel, 2016 (“sp. nov.”)

##### Notes

One new species was described (Tautel 2016).

#### Chlorochromodes

Warren, 1896

AA16DB10-4163-5DC0-BC5D-6E4F26B9FAAA

Comostolodes Warren, 1896 (“syn. nov.”)
Chlorochromodes albicatena (Warren, 1896) (“comb. nov.”)
Chlorochromodes chlorochromodes (Prout, 1916) (“comb. nov.”)
Chlorochromodes dialitha (West, 1930) (“comb. nov.”)
Chlorochromodes rhodocraspeda Han, Galsworthy & Xue, 2012 (“sp. nov.”)
Chlorochromodes tenera (Warren, 1896) (“comb. nov.”)
Chlorochromodes tumona Tautel, 2016 (“sp. nov.”)

##### Notes

Two new species were described (Han et al. 2012, Tautel 2016). *Comostolodes* was designated a junior synonym of *Chlorochromodes* by Han et al. (2012), who consequently formed new combinations for four species formerly in *Comostolodes*.

#### Chloroglyphica

Warren, 1894

EB5AEAF1-C436-5EA5-8172-D2A3E98F5277

Chloroglyphica glaucochrista (Prout, 1916)
Chloroglyphica glaucochrista grearia (Oberthür, 1916) (“syn. nov.”)

##### Notes

The status of *Chloroglyphica glaucochrista grearia* was changed from subspecies to junior synonym of *C. glaucochrista* by Han and Xue (2011a).

#### Chlororithra

Butler, 1889

1793074D-B595-5AF3-9745-E66731F73377

Chlororithra fea Butler, 1889
Chlororithra missioniaria Oberthür, 1916 (“stat. nov.”)

##### Notes

*Chlororithra missioniaria* was originally described as a variation of *C. fea* by Oberthür (1916). Parsons et al. (1999) instead treated *C. missioniaria* as a junior synonym of *C. fea*, so the name was absent from the checklist of Scoble and Hausmann (2007); however, prior to the publication of the checklist, *C. missioniaria* was designated a distinct species of *Chlororithra* by Han et al. (2006).

#### Comibaena

Hübner, [1823]

DFDDA539-4486-55E6-92B5-BE2B4274B0CA

Comibaena auromaculata Han, Galsworthy & Xue, 2012 (“sp. nov.”)
Comibaena bellula Han, Galsworthy & Xue, 2012 (“sp. nov.”)
Comibaena birectilinea Han, Galsworthy & Xue, 2012 (“sp. nov.”)
Comibaena decora Han, Galsworthy & Xue, 2012 (“sp. nov.”)
Comibaena levequei Leraut, 2009 (“sp. nov.”)
Comibaena nigromacularia (Leech, 1897)
Comibaena delicatior (Warren, 1897) (“syn. nov.”)
Comibaena parornataria Han, Galsworthy & Xue, 2012 (“sp. nov.”)
Comibaena pictipennis Butler, 1880
Comibaena pictipennis superornataria (Oberthür, 1916) (“syn. nov.”)
Comibaena sheni Han, Galsworthy & Xue, 2012 (“sp. nov.”)
Comibaena tibetensis Han, Galsworthy & Xue, 2012 (“sp. nov.”)
Comibaena theodori Hausmann & Parisi, 2014 (“sp. nov.”)

##### Notes

Nine new species were described (Han et al. 2012, Hausmann et al. 2014).

Müller et al. (2019) noted that *Comibaena levequei* may be identical to *C. pseudoneriaria* Wehrli, 1926, but tentatively accepted it as a distinct species. *Comibaena delicatior* was synonymised with *C. nigromacularia* by Han and Xue (2011a). *Comibaena pictipennis superornataria* had its status changed from subspecies to junior synonym of *C. pictipennis* by Han et al. (2012).

#### Comostola

Meyrick, 1888

8D8DC79F-0D7A-525B-9DE7-FF3ED61ABDBA

Comostola christinaria (Oberthür, 1916) (“comb. nov.”)
Comostola desdemona Tautel, 2015 (“sp. nov.”)
Comostola romblonensis Tautel & Barrion-Dupo, 2017 (“sp. nov.”)
Comostola stueningi Tautel & Barrion-Dupo, 2017 (“sp. nov.”)

##### Notes

Three new species were described (Tautel 2015, Tautel and Barrion-Dupo 2017). *Comostola christinaria* was transferred from *Hemistola* Warren, 1893 by Han and Xue (2009).

#### Crypsiphona

Meyrick, 1888

E9D7EDCA-D804-52FB-865E-F31C462128CB

Crypsiphona tasmanica Õunap & Viidalepp, 2009 (“sp. nov.”)

##### Notes

One new species was described (Õunap and Viidalepp 2009).

#### Dindica

Moore, 1888

2D7F92BF-5C96-56F6-B57E-3B29AB0D28D5

Dindica purpurata Bastelberger, 1911
Dindica wytsmani Prout, 1927 (“stat. rev.”)

##### Notes

*Dindica purpurata wytsmani* was elevated from subspecies to species by Pitkin et al. (2007).

#### Dindicodes

Prout, 1912

8C8C8446-529E-5089-AC96-6874A2DBEB7F

Dindicodes albodavidaria (Xue, 1992) (“comb. nov.”)
Dindicodes apicalis (Moore, 1888)(“comb. rev.”)
Dindicodes apicalis hunana (Xue, 1992) (“comb. nov.”)
Dindicodes costiflavens (Wehrli, 1933) (“comb. nov.”)
Dindicodes davidaria (Poujade, 1895) (“comb. rev.”)
Dindicodes ectoxantha (Wehrli, 1933) (“comb. nov.”)
Dindicodes euclidiaria (Oberthür, 1913) (“comb. rev.”)
Dindicodes harutai (Yazaki, 1992) (“comb. nov.”)
Dindicodes harutai infuscatus (Yazaki, 1992) (“comb. nov.”)
Dindicodes leopardinata (Moore, 1868) (“comb. rev.”)
Dindicodes moelleri (Warren, 1893) (“comb. rev.”)

##### Notes

The 11 species and subspecies listed here were formally transferred from the genus *Pachyodes* Guenée, [1858] to *Dindicodes* by Pitkin et al. (2007).

#### Dioscore

Warren, 1907

D51FF1E8-38D5-590A-8089-9AD4A147BA09

Dioscore kirke Lindt, Lennuk & Viidalepp, 2017 (“sp. nov.”)
Dioscore vilu Lindt, Lennuk & Viidalepp, 2017 (“sp. nov.”)

##### Notes

Two new species were described (Lindt et al. 2017a).

#### Dysphania

Hübner, [1819]

155808DB-13A3-59B9-81E5-78B59BE26E6F

Dysphania discalis aureolina Inoue, 2007 (“ssp. nov.”)

##### Notes

One new species was described (Inoue 2007).

#### Epichrysodes

Han & Stüning, 2007 (“gen. nov.”)

CBC0752C-7CB1-574E-B934-20BAD011D9F9

Epichrysodes tienmuensis Han & Stüning, 2007 (“sp. nov.”)

##### Notes

*Epichrysodes* is currently a monotypic genus containing only the type species, *E. tienmuensis*; both the genus and the species were described by Han et al. (2007).

#### Epipristis

Meyrick, 1888

2E229E94-55BF-570E-9008-E4B9E7876E9F

Epipristis pullusa Han & Xue, 2009 (“sp. nov.”)
Epipristis roseus Expósito & Han, 2009 (“sp. nov.”)

##### Notes

Two new species were described (Han et al. 2009a).

#### Episothalma

Swinhoe, 1893

D3F8A63B-077B-51A5-9956-985002CFDF6D

Episothalma cuspidata Xue & Wang, 2009 (“sp. nov.”)
Episothalma irrobustaria Xue & Wang, 2009 (“sp. nov.”)

##### Notes

Two new species were described (Xue et al. 2009).

#### Eucyclodes

Warren, 1894

783E4343-F800-588F-B51C-A5704DFCD722

Eucyclodes aphrodite (Prout, 1933) (“stat. nov.”)
Eucyclodes gavissima (Walker, 1861)
Eucyclodes hiyasata Tautel, 2016 (“sp. nov.”)
Eucyclodes insolita Han & Zhang, 2019 (“sp. nov.”)
Eucyclodes omeica (Chu, 1981) (“comb. nov.”)

##### Notes

Two new species were described (Tautel 2016, Zhang et al. 2019). Han and Xue (2011a) elevated *Eucyclodes gavissima aphrodite* (Prout, 1933) from subspecies to species and transferred *Chloromachia omeica* Chu, 1981 to *Eucyclodes*. *Chloromachia* was already considered a junior synonym of *Eucyclodes* (Parsons et al. 1999), but Han and Xue (2011a) were the first to formally publish the new combination *E. omeica*.

#### Geometra

Linnaeus, 1758

973CBEA8-F5B6-5FA6-8E95-910C359EAD44

Geometra neovalida Han, Galsworthy & Xue, 2009 (“sp. nov.”)

##### Notes

One new species was described (Han et al. 2009b).

#### Gnophosema

Prout, 1912

5C4211BE-ABB7-55FC-9F10-A36074437F07

Gnophosema isometra (Warren, 1888)
Gnophosema leucites Wiltshire, 1980 (“stat. nov.”)

##### Notes

*Gnophosema isometra leucites* Wiltshire, 1980 was elevated from subspecies to species by Hausmann (2009).

#### Haruchlora

Viidalepp & Lindt, 2014 (“gen. nov.”)

3C5E68CB-85C5-519A-B31E-C21D13C33821

Haruchlora maesi Viidalepp & Lindt, 2014 (“sp. nov.”)

##### Notes

*Haruchlora* is currently a monotypic genus containing only the type species, *H. maesi*; both the genus and the species were described by Viidalepp and Lindt (2014).

#### Hemistola

Warren, 1893

F2412824-074D-5751-B1BB-9345D1FF44E6

Hemistola arcilinea Han & Xue, 2009 (“sp. nov.”)
Hemistola asymmetra Han & Xue, 2009 (“sp. nov.”)
Hemistola flavifimbria Han & Xue, 2009 (“sp. nov.”)
Hemistola flavitincta Warren, 1897 (“comb. rev.”)
Hemistola fui Chang & Wu, 2013 (“sp. nov.”)
Hemistola glauca Han & Xue, 2009 (“sp. nov.”)
Hemistola hanae Wu, 2019 (“sp. nov.”)
Hemistola liliana (Swinhoe, 1892) (“comb. rev.”)
Hemistola orbiculosoides Han & Xue, 2009 (“sp. nov.”)
Hemistola piceacola Chang & Wu, 2013 (“sp. nov.”)
Hemistola stueningi Han & Xue, 2009 (“sp. nov.”)
Hemistola taiwanensis Chang & Wu, 2013 (“sp. nov.”)
Hemistola viridimargo Han & Xue, 2009 (“sp. nov.”)

##### Notes

Eleven new species were described (Han and Xue 2009, Chang and Wu 2013, Wu 2019). The species *Hemistola flavitincta* and *Hemistola liliana* were transferred to *Herochroma* Swinhoe, 1893 by Parsons et al. (1999). Pitkin et al. (2007) implied this was an editorial error and, citing a complete absence of *Herochroma* diagnostic characters, transferred both species back to *Hemistola*.

#### Hemithea

Duponchel, 1829

2A8D74AB-0726-52B5-BA88-A1508DF3CC5E

Hemithea aestivaria (Hübner, 1789)
Hemithea aestivaria alboundulata (Hedemann, 1879) (“stat. nov.”, followed by “syn. rev.”)

##### Notes

*Hemithea alboundulata* was a junior synonym of *H. aestivaria* until Leraut (2009) elevated it to subspecies. Müller et al. (2019) found the justification for this taxonomic change to be too vague and, consequently, revived its status as a synonym of *H. aestivaria*.

#### Herochroma

Swinhoe, 1893

D9460764-45AA-57E9-98D7-1ACEBC8FA45A

Herochroma costata Kirti, Goyal & Kaur, 2012 (nom. nud.)
Herochroma subspoliata (Prout, 1916)
Herochroma xuthopletes (Prout, 1934) (“stat. rev.”)

##### Notes

The name and locality of *Herochroma costata* were published in Kirti et al. (2012), but its description and diagnosis can only be found in the first author’s unpublished thesis. This species name is thus considered a nomen nudum.

*Herochroma subspoliata xuthopletes* (Prout, 1934) was elevated from subspecies to species by Pitkin et al. (2007).

#### Hypobapta

Prout, 1912

68E712F1-8BA7-5CB4-BFE5-D7173EF3E278

Hypobapta tachyhalotaria Hausmann & Sommerer, 2009 (“sp. nov.”)

##### Notes

One new species was described (Hausmann et al. 2009).

#### Jodis

Hübner, [1823]

AF1E1F69-36A4-587F-9F53-2298F123D086

Jodis altitudinis Tautel, 2016 (“sp. nov.”)
Jodis argentea Tautel, 2016 (“sp. nov.”)
Jodis berde Tautel, 2016 (“sp. nov.”)
Jodis mystica Tautel, 2016 (“sp. nov.”)
Jodis omeiensis (Chu, 1981) (“comb. nov.”)
Jodis orientalis Wehrli, 1923 (“stat. nov.”)
Jodis angulata Inoue, 1961 (“syn. nov.”)
Jodis putata (Linnaeus, 1758)
Jodis sibuyana Tautel, 2016 (“sp. nov.”)
Jodis tomopunctata Tautel, 2016 (“sp. nov.”)

##### Notes

Six new species were described (Tautel 2016).

Han and Xue (2011a) created the new combination *Jodis omeiensis*, stating that this species was transferred from the genus *Gelasma* Warren, 1893; however, *Gelasma* had been designated a junior synonym of *Maxates* Moore, [1887] by Holloway (1996). Despite this synonymy, the combination *Maxates omeiensis* (Chu, 1981) does not appear to have ever been published between 1996 and 2011.

Beljaev (2007) elevated *Jodis putata orientalis* Wehrli, 1923 from subspecies to species and subsequently synonymised it with *J. angulata*.

#### Kuchleria

Hausmann, 1995

5E3BE2C5-B3A0-5AE1-ADB2-57CDC3144125

Kuchleria menadiara Thierry-Mieg, 1893
Kuchleria insignata Hausmann, 1995 (“syn. nov.”, followed by “stat. rev.”)
Kuchleria garciapitai Expósito, 2006 (“syn. nov.”)

##### Notes

*Kuchleria garciapitai* was designated a junior synonym of *K. insignata* by Leraut (2009). In the same publication, Leraut (2009) claimed that *K. insignata* was a “synonym or subspecies” of *Kuchleria menadiara* Thierry-Mieg, 1893. Müller et al. (2019) treated this claim as a formal synonymy of *K. insignata* and *K. menadiara* and, subsequently, provided molecular and morphological evidence to justify elevating it back to species. *Kuchleria garciapitai* remains a junior synonym of *K. insignata*.

#### Lindachlora

Viidalepp & Lindt, 2012 (“gen. nov.”)

161A4BBD-6804-51B6-93BC-00BFEDBA4B32

Lindachlora flaccida (Warren, 1909) (“comb. nov.”)
Lindachlora tanystys (Prout, 1931) (“comb. nov.”)

##### Notes

The genus *Lindachlora* currently contains two species, both of which were transferred from *Phrudocentra* Warren, 1895 by Viidalepp and Lindt (2012), with *L. flaccida* designated as the type species.

#### Linguisaccus

Han, Galsworthy & Xue, 2012 (“gen. nov.”)

15BDF026-E5B7-524B-AEEB-91C24C41144E

Linguisaccus minor Han, Galsworthy & Xue, 2012 (“sp. nov.”)
Linguisaccus subhyalina (Warren, 1899) (“comb. nov.”)

##### Notes

*Comostolodes subhyalina* Warren, 1899 was transferred to *Comibaena* by Han and Xue (2011a) and then designated as the type species of the new genus *Linguisaccus* by Han et al. (2012).

#### Lissocentra

Viidalepp & Lindt, 2012 (“gen. nov.”)

077F3EC9-8A2D-5FEE-9898-F4102DA55192

Lissocentra hydatodes (Warren, 1906) (“comb. nov.”)
Lissocentra vitiosaria (Dognin, 1912) (“comb. nov.”)

##### Notes

The recently described genus *Lissocentra* currently contains two species, both of which were transferred from *Phrudocentra* by Viidalepp and Lindt (2012), with *L. hydatodes* designated as the type species.

#### Lissochlora

Warren, 1900

F1DB66A7-E0A0-51F3-83B0-69F72E5CAD7D

Lissochlora hinojosae Lindt & Viidalepp, 2014 (“sp. nov.”)
Lissochlora janamariae Lindt & Viidalepp, 2014 (“sp. nov.”)
Lissochlora klausi Viidalepp & Lindt, 2019 (“sp. nov.”)
Lissochlora niveiceps (Prout, 1912) (“comb. nov.”)
Lissochlora senescens (Prout, 1917) (“comb. nov.”)

##### Notes

Three new species were described (Lindt and Viidalepp 2014, Viidalepp and Lindt 2019a). One of those species, *Lissochlora hinojosae*, was described in Lindt et al. (2014) with the specific epithet spelled ‘*hinoyosae*’, the first time it appears in both the English and Spanish versions of the abstract. However, it is spelled ‘*hinojosae*’ the first time it appears in the main text. The etymological remarks provided in Lindt et al. (2014) confirm that ‘*hinojosae*’ is the intended spelling (cf. § 24.2; 32.2.1.; 32.5 Code ICZN).

*Lissochlora niveiceps* and *L. senescens* were transferred from *Phrudocentra* by Viidalepp and Lindt (2012).

#### Lophophelma

Prout, 1912

0EADF3D8-3F8D-55FF-879D-5BDD6E10B7C9

Lophophelma albapex (Inoue, 1988) (“comb. nov.”)
Lophophelma costistrigaria (Moore, 1868) (“comb. rev.”)
Lophophelma iterans (Prout, 1926) (“comb. nov.”)
Lophophelma pingbiana (Chu, 1981) (“comb. nov.”)
Lophophelma taiwana (Wileman, 1912) (“comb. rev.”)
Lophophelma tanatoraja Sommerer, Stüning & Tautel, 2015 (“sp. nov.”)
Lophophelma varicoloraria (Moore, 1868) (“comb. rev.”)

##### Notes

One new species was described (Sommerer et al. 2015). Pitkin et al. (2007) transferred six species to *Lophophelma*: five from the genus *Pachyodes* (*Lophophelma albapex*, *L. costistrigaria*, *L. iterans*, *L. taiwana*, *L. varicoloraria*) and one from the genus *Terpna* Herrich-Schäffer, 1854 (*Lophophelma pingbiana*).

#### Loxochila

Butler, 1881 (“stat. rev.”)

FF7B47F1-DFD7-5903-A101-67B790385CF7

Loxochila burmensis (Han, Galsworthy & Xue, 2009) (“sp. nov.”)
Loxochila fragilis (Oberthür, 1916) (“comb. nov.”)
Loxochila kina (Swinhoe, 1893) (“comb. nov.”)
Loxochila sinoisaria (Oberthür, 1916) (“comb. nov.”)
Loxochila smaragdus (Butler, 1880) (“comb. rev.”)
Loxochila tibeta (Chu, 1982) (“comb. nov.”)

##### Notes

One new species was described (Han et al. 2009b). *Loxochila* was treated as a junior synonym of *Geometra* in Parsons et al. (1999) and Scoble and Hausmann (2007). Han et al. (2009b)retained this classification in their revision of *Geometra*, which divided the genus into two species groups. The type species of *Loxochila*, at the time named *Geometra smaragdus* (Butler), served as the eponymous taxon of the *smaragdus* species group. The molecular phylogeny of Ban et al. (2018) demonstrated that the *smaragdus* group is a strongly-supported clade that also contains one species from a different genus (*Tanaorhinus kina* Swinhoe). Ban et al. (2018) consequently re-instated the generic status of *Loxochila* and transferred the species in the *smaragdus* group, including *T. kina*, to this genus.

#### Maxates

Moore, [1887]

91CBABDF-028B-5C2C-B99F-96F87A694805

Maxates acyra (Prout, 1935) (“comb. nov.”)
Maxates dissimulata (Walker, 1861)
Maxates semiprotrusa (Inoue, 1989) (“syn. nov.”)
Maxates elegante Tautel, 2015 (“sp. nov.”)
Maxates persona Tautel, 2016 (“sp. nov.”)
Maxates szechwanensis (Chu, 1981) (“comb. nov.”)

##### Notes

Two new species were described (Tautel 2015, Tautel 2016). *Maxates acyra* was transferred from *Hemistola* by Han and Xue (2009) and *M. szechwanensis* was transferred from *Jodis* by Han and Xue (2011a). Han and Xue (2011a) also synonymised *M. semiprotrusa* with *M. dissimulata*.

#### Metaterpna

Yazaki, 1992

26B7D94E-D38B-5B4D-8D16-CD7270FED1BF

Metaterpna batangensis Han & Stüning, 2016 (“sp. nov.”)

##### Notes

One new species was described (Jiang et al. 2016).

#### Microloxia

Warren, 1893

0BA906A5-C421-5608-AE53-B576A58BABD4

Microloxia aistleitneri Hausmann, 2009 (“sp. nov.”)
Microloxia chlorissoides (Prout, 1912) (“comb. nov.”)
Microloxia herbaria (Hübner, 1813)
Microloxia herbaria virideciliata (Bubacek, 1926) (“syn. nov.”, followed by “stat. rev.”)

##### Notes

One new species was described (Hausmann 2009).

After synonymising *Aoshakuna* and *Nipponogelasma*, Beljaev (2007) transferred *Nipponogelasma chlorissoides* (Prout, 1913) to *Microloxia*.

Leraut (2009) synonymised *Microloxia herbaria virideciliata* with *M. h. herbaria* (Hübner, 1813). Müller et al. (2019) cited molecular and morphological evidence to justify elevating *M. h. virideciliata* back to subspecies.

#### Nemoria

Hübner, 1818

17BD7106-FE9B-5A21-8D22-9C864392EF35

Nemoria “*Nemoria*” *erina* (Dognin, 1896)
Nemoria “*Nemoria*” *nigrisquama* (Dognin, 1904)
Nemoria yellowrosea Koçak & Kemal, 2008 (“nom. nov.”)
Nemoria albilineata Cassino, 1927

##### Notes

The molecular phylogeny of Murillo-Ramos et al. (2019) indicates that the current concept of *Nemoria* Hübner is polyphyletic and that *N. erina* (Dognin) and *N. nigrisquama* (Dognin) do not belong in *Nemoria*, though there is insufficient evidence to describe new genera or create new combinations for these two species. Brehm et al. (2019) consequently suggested that their generic names are listed in quotation marks, pending further taxonomic study.

In her revision of Neotropical *Nemoria*, Pitkin (1993) transferred *Lissochlora albilineata* Warren, 1909 to the genus *Nemoria*. This new combination, *Nemoria albilineata* (Warren, 1909) consequently became a senior homonym of the Texan species *Nemoria albilineata* Cassino, 1927. This homonymy went unnoticed for over a decade, until Koçak and Kemal (2008) designated a replacement name for the junior homonym.

#### Neochloroglyphica

Han & Skou, 2019 (“gen. nov.”)

5406FEB8-A06F-5FAC-B5AB-8D71C8F4B9A6

Neochloroglyphica perbella Han & Skou, 2019 (“sp. nov.”)

##### Notes

*Neochloroglyphica* is currently a monotypic genus containing only the type species, *N. perbella*; both the genus and the species were described by Han et al. (2019).

#### Neohipparchus

Inoue, 1944

B7A6CCD0-ADFE-5048-9EDE-28725116C25B

Neohipparchus maculata (Warren, 1897)
Chloroglyphica orhanti Herbulot, 1994 (“syn. nov.”)

##### Notes

*Chloroglyphica orhanti* was synonymised with *Neohipparchus maculata* by Han and Xue (2011a).

#### Neromia

Staudinger, 1898

4BFE5813-C386-5353-B8D6-628A27A548D3

Neromia integrata Hausmann, 2009 (“sp. nov.”)

##### Notes

One new species was described (Hausmann and Hebert 2009).

#### Oenospila

Swinhoe, 1892

E6A920AC-D4DD-556A-915A-D262FC9476C2

Oenospila sacculstrix Kirti, Goyal & Kaur, 2012 (nom. nud.)

##### Notes

The name and locality of this species were formally published in Kirti et al. (2012), but its description and diagnosis can only be found in the first author’s unpublished thesis. This species name is thus considered a nomen nudum.

#### Oospila

Warren, 1897

2A00E10E-950B-5BCB-AE2B-76C03B6F75D7

Oospila absaloni Lindt, Hausmann & Viidalepp, 2018 (“sp. nov.”)
Oospila agnetaforslundae Lindt, Hausmann & Viidalepp, 2018 (“nom. nov.”)
Oospila bifida Lindt, Hausmann & Viidalepp, 2018 (“sp. nov.”)
Oospila brehmi Lindt, Hausmann & Viidalepp, 2018 (“sp. nov.”)
Oospila bulava Lindt & Viidalepp, 2015 (“sp. nov.”)
Oospila cristae Lindt, Hausmann & Viidalepp, 2018 (“sp. nov.”)
Oospila ehakernae Lindt, Hausmann & Viidalepp, 2018 (“sp. nov.”)
Oospila falcata Lindt, Hausmann & Viidalepp, 2018 (“sp. nov.”)
Oospila imula Dognin, 1911 (“stat. nov.”)
Oospila loreenae Lindt, Hausmann & Viidalepp, 2018 (“sp. nov.”)
Oospila moseri Lindt, Hausmann & Viidalepp, 2018 (“sp. nov.”)
Oospila pallidaria boliviensis Lindt, Hausmann & Viidalepp, 2018 (“ssp. nov.”)
Oospila pipa Lindt, Hausmann & Viidalepp, 2018 (“sp. nov.”)
Oospila poirieri Lévêque & Viidalepp, 2015 (“sp. nov.”)
Oospila similiplaga Warren, 1900 (“stat. nov.”)
Oospila similiplaga bolarpata Lindt, Hausmann & Viidalepp, 2018 (“ssp. nov.”)

##### Notes

Eleven species and two subspecies were described (Lévêque and Viidalepp 2015, Lindt and Viidalepp 2015, Lindt et al. 2018). Lindt et al. (2018) designated *Oospila agnetaforslundae* as a replacement name for *Oospila marginata* (Schaus, 1912), which had previously been erroneously synonymised with *Oospila permagna* (Warren, 1909) by Cook and Scoble (1995). The replacement name was necessary because *O. marginata* (Schaus, 1912) is a junior secondary homonym of *O. marginata* Warren, 1897. Lindt et al. (2018) also raised *O. imula* from synonymy with *O. miccularia* Guenée, [1858] and raised *O. similiplaga* from synonymy with *O. arpata* (Schaus, 1897).

#### Ornithospila

Warren, 1894

D7F00D60-3B82-5822-B539-45D4CE04A831

Ornithospila explorator Tautel, 2015 (“sp. nov.”)

##### Notes

One new species was described (Tautel 2015).

#### Orothalassodes

Holloway, 1996

FC69FB22-34AE-53F4-B1A6-6EF1EDBE4FA4

Orothalassodes leucospilota (Moore, [1887])
Thalassodes albomaculata Hampson, 1895
Orothalassodes albomaculata Kirti, Goyal & Kaur, 2012

##### Notes

Kirti et al. (2012) published the name *Orothalassodes albomaculata* as a new combination for *Thalassodes albomaculata*. However, *T. albomaculata* had already been synonymised with *Thalassodes leucospilota* Moore by Hampson (1896), which was then transferred to *Orothalassodes* by Holloway (1996). Thus, the current valid name for this species is still *Orothalassodes leucospilota*.

#### Pachyodes

Guenée, [1858]

02FB8F9B-6AA6-55CC-91E8-16663A5DED85

Pachyodes jianfengensis Han & Xue, 2008 (“sp. nov.”)
Pachyodes novata Han & Xue, 2008 (“sp. nov.”)

##### Notes

Two new species were described (Han and Xue 2008).

#### Paramaxates

Warren, 1894

FB500918-2293-5552-9223-0C94EA9CD198

Paramaxates fournieri Tautel, 2016 (“sp. nov.”)
Paramaxates vagata (Walker, 1861)
Paramaxates hainana Chu, 1981 (“syn. nov.”)

##### Notes

One new species was described (Tautel 2016). *Paramaxates hainana* was synonymised with *P. vagata* by Han and Xue (2011a).

#### Paromphacodes

Warren, 1897

FF04FAD8-1439-5E54-8D70-DF049656AE5A

Paromphacodes alpha Lindt, Tasane, Õunap & Viidalepp, 2017 (“sp. nov.”)
Paromphacodes alticola Lindt, Tasane, Õunap & Viidalepp, 2017 (“sp. nov.”)
Paromphacodes onae Lindt, Tasane, Õunap & Viidalepp, 2017 (“sp. nov.”)
Paromphacodes spina Lindt, Tasane, Õunap & Viidalepp, 2017 (“sp. nov.”)
Paromphacodes summita Lindt, Tasane, Õunap & Viidalepp, 2017 (“sp. nov.”)

##### Notes

Five new species were described (Lindt et al. 2017b).

#### Pelagodes

Holloway, 1996

67CE21E4-8201-5BCA-97FC-F20482D03AC4

Pelagodes bellula Han & Xue, 2011 (“sp. nov.”)
Pelagodes cancriformis Viidalepp, Han & Lindt, 2012 (“sp. nov.”)
Pelagodes paraveraria Han & Xue, 2011 (“sp. nov.”)
Pelagodes simplvalvae Han & Xue, 2011 (“sp. nov.”)
Pelagodes sinuspinae Han & Xue, 2011 (“sp. nov.”)

##### Notes

Five new species were described (Han and Xue 2011b, Viidalepp et al. 2012).

#### Prasinocyma

Warren, 1897

337CD292-D817-5E68-9287-8B40CC377A16

Prasinocyma amharensis Hausmann, Sciarretta & Parisi, 2016 (“sp. nov.”)
Prasinocyma angolica pseudopedicata Hausmann, Sciarretta & Parisi, 2016 (“ssp. nov.”)
Prasinocyma angolica yemenicola Hausmann & Wildfeuer, 2017 (“ssp. nov.”)
Prasinocyma angulifera Hausmann, Sciarretta & Parisi, 2016 (“sp. nov.”)
Prasinocyma aquamarina Hausmann, Sciarretta & Parisi, 2016 (“sp. nov.”)
Prasinocyma batesi distans Hausmann, Sciarretta & Parisi, 2016 (“ssp. nov.”)
Prasinocyma baumgaertneri Hausmann, Sciarretta & Parisi, 2016 (“sp. nov.”)
Prasinocyma beryllaria Hausmann, Sciarretta & Parisi, 2016 (“sp. nov.”)
Prasinocyma bongaensis Hausmann, Sciarretta & Parisi, 2016 (“sp. nov.”)
Prasinocyma camerunalta (Herbulot, 1986) (“comb. nov.”)
Prasinocyma discipuncta Hausmann, Sciarretta & Parisi, 2016 (“sp. nov.”)
Prasinocyma fallax Hausmann, Sciarretta & Parisi, 2016 (“sp. nov.”)
Prasinocyma fusca Hausmann, Sciarretta & Parisi, 2016 (“sp. nov.”)
Prasinocyma gemmifera Hausmann, Sciarretta & Parisi, 2016 (“sp. nov.”)
Prasinocyma getachewi Hausmann, Sciarretta & Parisi, 2016 (“sp. nov.”)
Prasinocyma immaculata (Thunberg, 1784)
Prasinocyma unipuncta Warren, 1897 (“syn. nov.”)
Prasinocyma immaculata thiaucourti Herbulot, 1993 (“stat. nov.”)
Prasinocyma leveneorum Hausmann, Sciarretta & Parisi, 2016 (“sp. nov.”)
Prasinocyma lutulenta Hausmann, Sciarretta & Parisi, 2016 (“sp. nov.”)
Prasinocyma magica Hausmann, Sciarretta & Parisi, 2016 (“sp. nov.”)
Prasinocyma monikae Hausmann, Sciarretta & Parisi, 2016 (“sp. nov.”)
Prasinocyma nereis Townsend, 1952 (“comb. rev.”)
Prasinocyma pedicata aethiopica Hausmann, Sciarretta & Parisi, 2016 (“ssp. nov.”)
Prasinocyma robusta Hausmann, Sciarretta & Parisi, 2016 (“sp. nov.”)
Prasinocyma saba Hausmann & Wildfeuer, 2017 (“sp. nov.”)
Prasinocyma septentrionalis Hausmann, Sciarretta & Parisi, 2016 (“sp. nov.”)
Prasinocyma shoa yabellensis Hausmann, Sciarretta & Parisi, 2016 (“ssp. nov.”)
Prasinocyma stefani Hausmann, Sciarretta & Parisi, 2016 (“sp. nov.”)
Prasinocyma trematerrai Hausmann, Sciarretta & Parisi, 2016 (“sp. nov.”)
Prasinocyma trematerraisimienensis Hausmann, Sciarretta & Parisi, 2016 (“ssp. nov.”)

##### Notes

Twenty new species and six new subspecies were described (Hausmann et al. 2016, Hausmann and Wildfeuer 2017). Hausmann et al. (2016) transferred *Thalassodes camerunalta* Herbulot, 1986 and *Eretmopus nereis* (Townsend, 1952) to the genus *Prasinocyma*. They also synonymised *P. unipuncta* with *P. immaculata* and changed the status of *P. thiaucourti* Herbulot, 1993 from a species to a subspecies of *P. immaculata*.

#### Protuliocnemis

Holloway, 1996

CFDFDB2F-508D-5D3D-B548-0E1EFFC0706F

Protuliocnemis candida Han, Galsworthy & Xue, 2012 (“sp. nov.”)
Protuliocnemis dissimilis Han, Galsworthy & Xue, 2012 (“sp. nov.”)
Protuliocnemis falcipennis (Yazaki, 1991) (“comb. nov.”)

##### Notes

Two new species were described (Han et al. 2012). *Protuliocnemis falcipennis* was transferred from the genus *Comibaena* by Han and Xue (2011a).

#### Pseudepisothalma

Han, 2009 (“gen. nov.”)

4DC4C94E-94BF-5F8E-8D1D-B9E8F100263C

Pseudepisothalma ocellata (Swinhoe, 1893) (“comb. nov.”)

##### Notes

*Pseudepisothalma* is currently a monotypic genus containing only the type species, *P. ocellata*, which was transferred from the genus *Episothalma*. The new genus description and new combination were presented in Xue et al. (2009), though only the third author (Han) is credited with authorship.

#### Psilotagma

Warren, 1894

119C2CF0-1DE0-559B-9323-23DAB825698B

Psilotagma pictaria (Moore, 1888) (“comb. nov.”)

##### Notes

*Psilotagma pictaria* was transferred from the genus *Pachyodes* by Pitkin et al. (2007).

#### Pyrochlora

Warren, 1895

349190BC-3B3C-5E9F-9124-1B73A2BB301D

Pyrochlora kuklase Viidalepp, 2009 (“sp. nov.”)
Pyrochlora motilonia Viidalepp, 2009 (“sp. nov.”)
Pyrochlora vogli Viidalepp, 2009 (“sp. nov.”)

##### Notes

Three new species were described (Viidalepp 2009).

#### Rhanidopsis

West, 1930

EBEC8DAF-08AF-5631-B81A-6D69D42A6000

Rhanidopsis kogeri Viidalepp & Lindt, 2010 (“sp. nov.”)

##### Notes

One new species was described (Viidalepp and Lindt 2010).

#### Rhuma

Walker, 1860

CC00412B-937B-59EE-A03D-60417CD5D7BE

Sterictopsis Warren, 1898 (“syn. nov.”)
Oxyphanes Turner, 1936 (“syn. nov.”)
Rhuma argyraspis (Lower, 1893) (“comb. nov.”)
Rhuma divergens (Goldfinch, 1929) (“comb. nov.”)
Rhuma thiobapta (Turner, 1936)(“comb. nov.”)

##### Notes

Pitkin et al. (2007) designated *Sterictopsis* and *Oxyphanes* as junior synonyms of *Rhuma* and consequently transferred *S. argyraspis* (Lower, 1893), *S. divergens* Goldfinch, 1929 and *O. thiobapta* Turner, 1936 to this genus.

#### Tachyphyle

Butler, 1881

D2C21DFB-4AB7-5212-962E-A5F4C0B1F727

Tachyphyle nielseni Viidalepp & Lindt, 2017 (“sp. nov.”)
Tachyphyle selini Viidalepp & Lindt, 2017 (“sp. nov.”)

##### Notes

Two new species were described (Viidalepp and Lindt 2017).

#### Tanaorhinus

Butler, 1879

C61601D3-1139-529B-98A9-7008914E9C2C

Tanaorhinus baruensis Orhant, 2014 (“sp. nov.”)
Tanaorhinus guitinguensis Tautel, 2014 (“sp. nov.”)
Tanaorhinus sultan Tautel, 2014 (“sp. nov.”)

##### Notes

Three new species were described (Orhant 2014, Tautel 2014).

#### Telotheta

Warren, 1900

BC719B1C-FD28-5550-AE1E-1070F2AF50DD

Telotheta fresei Lindt & Viidalepp, 2014 (“sp. nov.”)
Telotheta unoi Lindt & Viidalepp, 2014 (“sp. nov.”)

##### Notes

Two new species were described (Lindt and Viidalepp 2014).

#### Thalera

Hübner, [1823]

1DD62E31-8E9E-5D9C-902F-54B709D18EC9

Hethemia Ferguson, 1969 (“syn. nov.”)
Thalera pistasciaria (Guenée, 1858) (“comb. nov.”)

##### Notes

*Hethemia* sensu Ferguson was a monotypic genus, containing only the type species *H. pistasciaria*. Ban et al. (2018) provided morphological and molecular evidence to justify the designation of *Hethemia* as a junior synonym of *Thalera*, creating the new combination *T. pistasciaria*.

#### Thetidia

Boisduval, 1840

21C772E9-3A1A-570A-BDFB-50480AB83FEB

Thetidia chlorophyllaria (Hedemann, 1879)
Thetidia pekingensis (Chu, 1981) (“comb. nov.”, followed by “syn. nov.”)

##### Notes

The name *Thetidia pekingensis* (Chu, 1981) was first published in Han and Xue (2011a); it was not designated a new combination, but since *Euchloris* Hübner, [1823] was already known to be a synonym of *Thetidia* (Parsons et al. 1999), Han and Xue (2011a) were presumably transferring *Euchloris pekingensis* Chu, 1981 to *Thetidia.* This was confirmed by Han et al. (2012), who subsequently synonymised *T. pekingensis* with *T. chlorophyllaria*.

#### Timandromorpha

Inoue, 1944

97240A09-A473-5F9E-9F46-293795562BD8

Timandromorpha inouei Stüning & Yazaki, 2008 (“sp. nov.”)
Timandromorpha pinratanai Stüning & Yazaki, 2008 (“sp. nov.”)
Timandromorpha wangi Stüning & Yazaki, 2008 (“sp. nov.”)
Timandromorpha xuedayongi Orhant, 2013 (“sp. nov.”)

##### Notes

Four new species were described (Stüning and Yazaki 2008, Orhant 2013).

#### Vallichlora

Viidalepp & Lindt, 2019 (“gen. nov.”)

B63E05DB-3604-557D-BAB1-C480C1CF4F08

Vallichlora rara Viidalepp & Lindt, 2019 (“sp. nov.”)
Vallichlora selva Viidalepp & Lindt, 2019 (“sp. nov.”)

##### Notes

Two new species were described (Viidalepp and Lindt 2019b).

#### Xenozancla

Warren, 1893

0AF7B21C-0814-5F7F-9644-4BD5258B2B2B

Yinchie Yang, 1978 (“syn. nov.”)
Xenozancla versicolor Warren, 1893
Yinchie zaohui Yang, 1978 (“syn. nov.”)

##### Notes

Han et al. (2008) synonymised *Yinchie zaohui* with *Xenozancla versicolor*. Since *Y. zaohui* was the type species of its genus, *Yinchie* was consequently designated a junior synonym of *Xenozancla*.

### Other species affiliated with Geometrinae

#### Pseudobiston pinratanai

Inoue, 1994

71E2CC97-856D-5829-B86F-147CC8FD6D34

##### Notes

*Pseudobiston pinratanai* Inoue, 1994, was classified as a geometrine in Scoble and Hausmann (2007) but was recently transferred to the new family Pseudobistonidae by Rajaei et al. (2015).

#### Cerura melanoglypta

(Lower, 1905)

0CF84C6D-3590-5713-9D32-CAF2CC432888

##### Notes

One species in the Scoble and Hausmann (2007) checklist, *Cerura melanoglypta* (Lower, 1905), is classified as a geometrine (Ollerenshaw 2012), but has never formally been transferred from the notodontid genus *Cerura* Schrank, 1802. We agree that this species should eventually be assigned to a genus in Geometrinae, but it is technically not in Geometrinae at this time.

## Discussion

In summation, nine new genera, 128 new species and ten new subspecies of emerald moths have been described since the publication of Scoble and Hausmann (2007), along with over 80 new genus- and species-group changes within subfamily Geometrinae. Since 2007, the known species richness of Geometrinae has increased by ~4.5%, from 2,529 species (Scoble and Hausmann 2007) to 2,643 species.

## Supplementary Material

EBEC8EED-8D51-5F90-9015-B64CDC4191C7 10.3897/BDJ.8.e52190.suppl1 Supplementary material 1 List of the Geometrinae species of the world Data type Taxonomical checklist Brief description This table provides a list of all new species descriptions, combinations and other taxonomic changes in the subfamily Geometrinae (Lepidoptera: Geometridae) since 2007 (Sheet 1: "Taxonomic changes since 2007"). This table also provides a list of the 2,643 current valid species names in the subfamily Geometrinae (Lepidoptera: Geometridae), with their authorship and year of description (Sheet 2: "All current Geometrinae species"). File: oo_400377.xls https://binary.pensoft.net/file/400377 David Plotkin

XML Treatment for Acidaliastis

XML Treatment for Agathia

XML Treatment for Albinospila

XML Treatment for Aoshakuna

XML Treatment for Assachlora

XML Treatment for Bathycolpodes

XML Treatment for Bustilloxia

XML Treatment for Chlorissa

XML Treatment for Chloristola

XML Treatment for Chlorochromodes

XML Treatment for Chloroglyphica

XML Treatment for Chlororithra

XML Treatment for Comibaena

XML Treatment for Comostola

XML Treatment for Crypsiphona

XML Treatment for Dindica

XML Treatment for Dindicodes

XML Treatment for Dioscore

XML Treatment for Dysphania

XML Treatment for Epichrysodes

XML Treatment for Epipristis

XML Treatment for Episothalma

XML Treatment for Eucyclodes

XML Treatment for Geometra

XML Treatment for Gnophosema

XML Treatment for Haruchlora

XML Treatment for Hemistola

XML Treatment for Hemithea

XML Treatment for Herochroma

XML Treatment for Hypobapta

XML Treatment for Jodis

XML Treatment for Kuchleria

XML Treatment for Lindachlora

XML Treatment for Linguisaccus

XML Treatment for Lissocentra

XML Treatment for Lissochlora

XML Treatment for Lophophelma

XML Treatment for Loxochila

XML Treatment for Maxates

XML Treatment for Metaterpna

XML Treatment for Microloxia

XML Treatment for Nemoria

XML Treatment for Neochloroglyphica

XML Treatment for Neohipparchus

XML Treatment for Neromia

XML Treatment for Oenospila

XML Treatment for Oospila

XML Treatment for Ornithospila

XML Treatment for Orothalassodes

XML Treatment for Pachyodes

XML Treatment for Paramaxates

XML Treatment for Paromphacodes

XML Treatment for Pelagodes

XML Treatment for Prasinocyma

XML Treatment for Protuliocnemis

XML Treatment for Pseudepisothalma

XML Treatment for Psilotagma

XML Treatment for Pyrochlora

XML Treatment for Rhanidopsis

XML Treatment for Rhuma

XML Treatment for Tachyphyle

XML Treatment for Tanaorhinus

XML Treatment for Telotheta

XML Treatment for Thalera

XML Treatment for Thetidia

XML Treatment for Timandromorpha

XML Treatment for Vallichlora

XML Treatment for Xenozancla

XML Treatment for Pseudobiston pinratanai

XML Treatment for Cerura melanoglypta

## References

[B5534990] Ban Xiaoshung, Jiang Nan, Cheng Rui, Xue Dayong, Han Hongxiang (2018). Tribal classification and phylogeny of Geometrinae (Lepidoptera: Geometridae) inferred from seven gene regions.. Zoological Journal of the Linnean Society.

[B5535761] Beljaev EA (2007). Taxonomic changes in the emerald moths (Lepidoptera: Geometridae, Geometrinae) of East Asia, with notes on the systematics and phylogeny of Hemitheini.. Zootaxa.

[B5535732] Brehm G., Murillo-Ramos L., Sihvonen P., Hausmann A, Schmidt BC, Õunap E, Moser A, Mörtter R, Bolt D, Bodner F, Lindt A, Parra L, Wahlberg N (2019). New World geometrid moths (Lepidoptera: Geometridae): Molecular phylogeny, biogeography, taxonomic updates and description of 11 new tribes.. Arthropod Systematics & Phylogeny.

[B5535771] Canfield Michael R., Greene Erick, Moreau Corrie S., Chen Nancy, Pierce Naomi E. (2008). Exploring phenotypic plasticity and biogeography in emerald moths: A phylogeny of the genus *Nemoria* (Lepidoptera: Geometridae). Molecular Phylogenetics and Evolution.

[B5535782] Chang W-C, Wu S (2013). Review of the genus *Hemistola* Warren, 1893 in Taiwan with notes on an unusual conifer-feeding larva and descriptions of three new species (Lepidoptera, Geometridae, Geometrinae). Zootaxa.

[B5535792] Cook MA, Scoble MJ (1995). Revision of the neotropical genus *Oospila* Warren (Lepidoptera: Geometridae).. Bulletin of the Natural History Museum. Entomology Series.

[B5535811] De Prins J., De Prins W. Afromoths, online database of Afrotropical moth species (Lepidoptera).. http://www.afromoths.net.

[B5729609] Goyal Tarun, Kirti Jagbir Singh, Saxena Abhinav (2018). Taxonomy of genus *Agathia* Guenée (Lepidoptera: Geometridae) with description of a new species from western ghats, India. Indian Journal of Entomology.

[B5535820] Greene E (1989). A diet-induced developmental polymorphism in a caterpillar. Science.

[B5535830] Hampson GF (1896). The fauna of British India, including Ceylon and Burma. Moths. Vol. IV..

[B5535859] Han H, Li H, Xue D (2006). Revision of *Chlororithra* Butler, 1889 (Lepidoptera, Geometridae, Geometrinae). Zootaxa.

[B5535899] Han Hongxiang, Stüning Dieter, Xue Dayong (2007). *Epichrysodes* gen. n., a new genus of Geometrinae from the West Tianmu mountains, China (Lepidoptera, Geometridae), with description of a new species. Mitteilungen aus dem Museum für Naturkunde in Berlin – Deutsche Entomologische Zeitschrift.

[B5535909] Han H, Xue D (2008). A taxonomic review of *Pachyodes* Guenée, 1858, with descriptions of two new species (Lepidoptera: Geometridae, Geometrinae).. Zootaxa.

[B5535879] Han H, Li J, Xue D (2008). Revision of the genus *Xenozancla* Warren, 1893 (Lepidoptera: Geometridae: Geometrinae) with an analysis of its distribution pattern.. Acta Entomologica Sinica.

[B5535919] Han Hongxiang, Xue Dayong (2009). Taxonomic review of *Hemistola* Warren, 1893 from China, with descriptions of seven new species (Lepidoptera: Geometridae, Geometrinae). Entomological Science.

[B5535869] Han H, Expósito-Hermosa A, Xue D (2009). A taxonomic study of *Epipristis* Meyrick, 1888 from China, with descriptions of two new species (Lepidoptera: Geometridae, Geometrinae). Zootaxa.

[B5535839] Han H‐X., Galsworthy A. C., Xue D‐Y. (2009). A survey of the genus *Geometra* Linnaeus (Lepidoptera, Geometridae, Geometrinae). Journal of Natural History.

[B5535929] Han H, Xue D (2011). Fauna Sinica (Insecta Vol. 54. Lepidoptera, Geometridae, Geometrinae)..

[B5535938] Han H, Xue D (2011). *Thalassodes* and related taxa of emerald moths in China (Geometridae, Geometrinae).. Zootaxa.

[B5535849] Han H, Galsworthy AC, Xue D (2012). The Comibaenini of China (Geometridae: Geometrinae), with a review of the tribe. Zoological Journal of the Linnean Society.

[B5535889] Han H, Skou P, Cheng R (2019). *Neochloroglyphica*, a new genus of Geometrinae from China (Lepidoptera, Geometridae), with description of a new species. Zootaxa.

[B5535948] Hausmann A (2009). New and interesting geometrid moths from the Cape Verde Islands (Lepidoptera: Geometridae).. SHILAP Revista de Lepidopterología.

[B5541611] Hausmann A., Hebert P. (2009). Order Lepidoptera, family Geometridae (Part 2). The Geometridae of the UAE revised in the light of mtDNA data.. Arthropod Fauna of the UAE.

[B5535978] Hausmann A, Sommerer M, Rougerie R, Hebert P (2009). *Hypobapta
tachyhalotaria* spec. nov from Tasmania – an example of a new species revealed by DNA barcoding.. Spixiana.

[B5535958] Hausmann A, Parisi F, Sciarretta A (2014). The geometrid moths of Ethiopia I: tribes Pseudoterpnini and Comibaenini (Lepidoptera: Geometridae, Geometrinae). Zootaxa.

[B5535968] Hausmann A, Sciarretta A, Parisi F (2016). The Geometrinae of Ethiopia II: Tribus Hemistolini, genus *Prasinocyma* (Lepidoptera: Geometridae, Geometrinae). Zootaxa.

[B5535988] Hausmann A, Wildfeuer J (2017). Nine new emerald species for the fauna of Yemen, with description of two new taxa in the genus *Prasinocyma.*. Spixiana.

[B5535998] Holloway JD (1996). The moths of Borneo: Family Geometridae, subfamiles Oenochrominae, Desmobathrinae and Geometrinae. Malayan Nature Journal.

[B5536008] Inoue H (2007). A new subspecies of *Dysphania
discalis* (Walker) (Geometridae, Geometrinae) from the Lingga Islands.. Lepidoptera Science.

[B5536018] Jiang N, Stüning D, Xue D, Han H (2016). Revision of the genus *Metaterpna* Yazaki, 1992 (Lepidoptera, Geometridae, Geometrinae), with description of a new species from China. Zootaxa.

[B5536028] Karisch T (2010). Geometridae der Expeditionen H. Hoppes nach Bioko 1. Teil: Desmobathrinae, Geometrinae (Lepidoptera, Geometridae).. Lambillionea.

[B5536792] Kirti JS, Goyal T, Kaur M (2012). An inventory of family Geometridae (Lepidoptera) from western ghats of India.. Journal of Entomological Research.

[B5537299] Kitching Ian, Rougerie Rodolphe, Zwick Andreas, Hamilton Chris, St Laurent Ryan, Naumann Stefan, Ballesteros Mejia Liliana, Kawahara Akito (2018). A global checklist of the Bombycoidea (Insecta: Lepidoptera). Biodiversity Data Journal.

[B5536802] Koçak AÖ, Kemal M. (2008). Some nomenclatural notes on the Geometridae of the world (Lepidoptera).. Miscellaneous papers - Centre for Entomological Studies.

[B5536812] Leraut P (2009). Moths of Europe-Volume 2: Geometrid moths..

[B5536821] Lévêque Antoine, Viidalepp Jaan (2015). Description of a new *Oospila* Warren, 1897, from French Guiana (Lepidoptera
Geometridae
Geometrinae
Lophochoristini). Antenor.

[B5536871] Lindt A, Viidalepp J (2014). Two new emerald geometrid species of *Telotheta* Warren from Ecuador and Bolivia (Lepidoptera: Geometridae, Geometrinae, Lophochoristini). Biodiversity Data Journal.

[B5536851] Lindt A, Tasane T, Viidalepp J (2014). Dos nuevas especies de polillas esmeraldas de Ecuador, Perú y Bolivia (Lepidoptera: Geometridae, Geometrinae).. SHILAP Revista de Lepidopterología.

[B5536881] Lindt A, Viidalepp J (2015). *Oospila
bulava*, a new emerald geometrid moth from South America (Lepidoptera, Geometridae, Geometrinae). Zootaxa.

[B5536841] Lindt Aare, Lennuk Lennart, Viidalepp Jaan (2017). The genus *Dioscore* Warren, 1907: two new species and analysis of characters spread (Lepidoptera: Geometridae: Geometrinae). Journal of Insect Biodiversity.

[B5536861] Lindt A, Tasane T, Õunap E, Viidalepp J (2017). Five new species of the genus *Paromphacodes* (Lepidoptera: Geometridae: Geometrinae) from High Andes in Ecuador. Zootaxa.

[B5536831] Lindt A, Hausmann A, Viidalepp J (2018). Review of some species groups of the genus *Oospila* Warren, with descriptions of nine new species (Lepidoptera: Geometridae: Geometrinae). Zootaxa.

[B5536891] Müller B, Erlacher S, Hausmann A, Rajaei H, Sihvonen P, Skou P (2019). Volume 6. Part 1. Subfamily Ennominae II: (Boarmiini, Gnophini, additions to previous volumes)..

[B5536902] Murillo-Ramos Leidys, Brehm Gunnar, Sihvonen Pasi, Hausmann Axel, Holm Sille, Reza Ghanavi Hamid, Õunap Erki, Truuverk Andro, Staude Hermann, Friedrich Egbert, Tammaru Toomas, Wahlberg Niklas (2019). A comprehensive molecular phylogeny of Geometridae (Lepidoptera) with a focus on enigmatic small subfamilies.. PeerJ.

[B5536920] Oberthür C (1916). Révision iconographique des espèces de Phalénites (Geometra, Linné).. Etudes de Lépidoptérologie Comparée.

[B5536930] Ollerenshaw J The Global Lepidoptera Names Index (LepIndex).. https://http://www.nhm.ac.uk/our-science/data/lepindex/detail/?taxonno=246329.

[B5536939] Orhant G (2013). Sept nouveaux hétérocères asiatiques (Lepidoptera, Noctuidae, Geometridae).. Lambillionea.

[B5536949] Orhant G (2014). Contribution à la connaissance du genre *Tanaorhinus* - Description d’une nouvelle espèce des Moluques. Découverte et description du mâle de *Tanaorhinus
tibeta* Chu, 1982 (Lepidoptera, Geometridae, Geometrinae).. Bulletin de la Société Entomologique de Mulhouse.

[B5536959] Õunap E, Viidalepp J (2009). Description of *Crypsiphona
tasmanica* sp. nov. (Lepidoptera: Geometridae: Geometrinae), with notes on limitations in using DNA barcodes for delimiting species.. Australian Journal of Entomology.

[B5536979] Parsons MS, Scoble MJ, Honey MR, Pitkin LM, Pitkin BR (1999). Geometrid moths of the world: a catalogue (Lepidoptera, Geometridae)..

[B5536989] Pitkin LM (1993). Neotropical emerald moths of the genera *Nemoria*, *Lissochlora* and *Chavarriella*, with particular reference to the species of Costa Rica (Lepidoptera: Geometridae, Geometrinae).. Bulletin of the Natural History Museum (Entomology).

[B5536999] Pitkin L, Han H, James S (2007). Moths of the tribe Pseudoterpnini (Geometridae: Geometrinae): a review of the genera. Zoological Journal of the Linnean Society.

[B5537009] Rajaei Hossein, Greve Carola, Letsch Harald, Stüning Dieter, Wahlberg Niklas, Minet Joël, Misof Bernhard (2015). Advances in Geometroidea phylogeny, with characterization of a new family based on *Pseudobiston
pinratanai* (Lepidoptera, Glossata). Zoologica Scripta.

[B5537022] Scoble M, Hausmann A Online list of valid and available names of the Geometridae of the World. http://www.lepbarcoding.org/geometridae/species_checklists.php.

[B5537041] Sommerer MD, Stüning D, Tautel C (2015). *Lophophelma
tanatoraja* spec. nov., a new geometrid moth from Sulawesi near *Lophophelma
luteipes* Felder & Rogenhofer, 1875 (Geometridae, Geometrinae).. Tinea.

[B5537051] Stüning D, Yazaki K (2008). Three new species of the genus *Timandromorpha* Inoue, 1944 (Lepidoptera, Geometridae, Geometrinae) from Southeast Asia.. Tinea.

[B5537061] Tautel C (2014). Deux nouveaux *Tanaorhinus* pour la Wallacea (Lepidoptera
Geometridae
Geometrinae).. Antenor.

[B5537071] Tautel C (2015). Nouveaux Geometridae des Philippines des genres *Comostola*, *Ornithospila* et *Maxates*.. Antenor.

[B5537081] Tautel C (2016). Nouvelles espèces de géomètres du Cambodge et des Philippines (Lepidoptera, Geometridae, Geometrinae). Bulletin de la Société Entomologique de France.

[B5537091] Tautel C, Barrion-Dupo A - L (2017). Nouvelles espèces de Geometrinae des Philippines dans les genres *Comostola* et *Albinospila* (Lepidoptera
Geometridae
Geometrinae).. Antenor.

[B5537121] van Nieukerken E. J., Kaila L, Kitching I J, Kristensen N P, Lees D C, Minet J, Mitter C, Mutanen M, Regier J C, Simonsen T J, Wahlberg N, Yen S - H, Zahiri R, Adamski D, Baixeras J, Bartsch D, Bengtsson B Å, Brown J W, Bucheli S R, Davis D R, De Prins J., De Prins W., Epstein M E, Gentili-Poole P, Gielis C, Hättenschwiler P, Hausmann A, Holloway J D, Kallies A, Karsholt O, Kawahara A Y, Koster S J C, Kozlov M, Lafontaine J D, Lamas G, Landry J F, Lee S, Nuss M, Park K T, Penz C, Rota J, Schmidt B C, Schintlmeister A, Sohn J C, Solis M A, Tarmann G M, Warren A D, Weller S, Yakovlev R V, Zolotuhin V V, Zwick A (2011). Order Lepidoptera Linnaeus, 1758. In: Zhang Z.-Q. (Ed) Animal biodiversity: An outline of higher-level classification and survey of taxonomic richness.. Zootaxa.

[B5537188] Viidalepp J (2009). Revision of the genus *Pyrochlora* Warren, 1895 (Lepidoptera: Geometridae: Geometrinae). Zootaxa.

[B5537198] Viidalepp J, Lindt A (2010). A new *Rhanidopsis* West, 1930 from the Malay Peninsula (Lepidoptera: Geometridae, Geometrinae).. SHILAP Revista de Lepidopterologia.

[B5537208] Viidalepp J, Lindt A (2012). A review of continental species of *Phrudocentra* Warren, 1895 (Lepidoptera: Geometridae, Geometrinae).. SHILAP Revista de Lepidopterología.

[B5537258] Viidalepp J, Lindt A, Han H (2012). *Pelagodes
cancriformis*, a new emerald moth species from the north of Thailand, Laos and southern China (Lepidoptera, Geometridae: Geometrinae). Zootaxa.

[B5537218] Viidalepp J, Lindt A (2014). *Haruchlora
maesi*, a new emerald moth genus and species from Mesoamerica (Lepidoptera, Geometridae, Geometrinae). Zootaxa.

[B5537228] Viidalepp J, Lindt A (2017). Two new species of *Tachyphyle* Butler, 1881 from South America (Lepidoptera: Geometridae).. SHILAP Revista de Lepidopterología.

[B5537238] Viidalepp J, Lindt A (2019). Description of a new species of the *Lissochlora
albociliaria* species group (Lepidoptera: Geometridae, Geometrinae). Zootaxa.

[B5537248] Viidalepp J, Lindt A (2019). A new Neotropical emerald moth genus based on some unusual “artefacts” (Lepidoptera: Geometridae, Geometrinae). Zootaxa.

[B5537268] Wu S (2019). Description of a new species of *Hemistola* Warren, 1893 from China (Geometridae: Geometrinae).. Tinea.

[B5537278] Xue D, Wang X, Han H (2009). A revision of *Episothalma* Swinhoe, 1893, with descriptions of two new species and one new genus (Lepidoptera, Geometridae, Geometrinae). Zootaxa.

[B5537288] Zhang X, Wang W, Han H (2019). A new species of the genus *Eucyclodes* (Lepidoptera, Geometridae, Geometrinae) from China. Zootaxa.

